# Roll-to-roll fabrication of silver/silver chloride coated yarns for dry electrodes and applications in biosignal monitoring

**DOI:** 10.1038/s41598-023-48245-8

**Published:** 2023-12-01

**Authors:** Katherine Le, Saeid Soltanian, Harishkumar Narayana, Amir Servati, Peyman Servati, Frank Ko

**Affiliations:** 1 Materials Engineering Department, University of British Columbia, Vancouver, BC V6T 1Z4 Canada; 2 Electrical and Computer Engineering Department, University of British Columbia, Vancouver, BC V6T 1Z4 Canada

**Keywords:** Biomedical materials, Characterization and analytical techniques

## Abstract

This work presents a continuous roll-to-roll electrochemical coating system for producing silver/silver chloride (Ag/AgCl)-coated yarns, and their application in e-textile electrodes for biosignal monitoring. Ag/AgCl is one of the most preferred electrode materials as an interface between the conductive backbone of an electrode and skin. E-textile Ag/AgCl-coated multi-filament nylon yarns offer stable, flexible, and breathable alternatives to standard rigid or flexible film-based Ag/AgCl electrodes. The developed system allows for highly controlled process parameters to achieve stable and uniform AgCl film deposition on Ag-coated nylon yarns. The electrical, electrochemical properties, and morphology of the coated yarns were characterized. Dry electrodes were fabricated and could measure electrocardiogram (ECG) signals with comparable performance to standard gel electrodes. Ag/AgCl e-textile electrodes demonstrated high stability, with low average polarization potential (1.22 mV/min) compared with Ag-coated electrodes (3.79 mV/min), low impedance (below 2 MΩ, 0.1–150 Hz), and are excellent candidates for heart rate detection and monitoring.

## Introduction

Biosignals can be detected and monitored from various locations of the body, by employing electrode sensors. Electrodes used for biosignal monitoring function by transducing ionic current (i.e., sodium, potassium, calcium, chloride ions) from the skin surface, into electron current, through metallic wires of a given recording system/instrument^1,2^.

Electrodes made from conductive metals or polymer films are commonly being used, but tend to have high sensitivity to motion artifacts, produce fluctuations in potential, and cause signal distortion over time, owing to the absence of an electrolyte layer^3^. However, sweat and moisture that develops on the skin surface over time is thought to act as an electrolyte layer, thereby reducing skin impedance^4^. While these distortions in features can be corrected by signal processing methods, it is more ideal to develop materials with polarization potential stability, to reduce interference with the collected signal^5,6^. Other considerations to be made when developing electrode materials for biosignal monitoring include low electrical resistance of the electrode compared with that for skin surface^7^, skin contact, conformability, and biocompatibility^8^.

Silver/Silver chloride (Ag/AgCl) electrode material is the gold standard for biosignal acquisition (electrocardiogram, electroencephalography, endodermal activity etc.), owing to its low and stable half-cell potential^2^. The most commonly used electrode for biosignal monitoring includes a pellet of sintered high purity silver and silver chloride, with a thin layer of conductive gel electrolyte coating on its top surface. This configuration provides good electric contact with the skin, and a metallic salt coating acts as an intermediate bridge between electrode to and electrolyte, thereby reducing skin–electrode impedance, which translates to good signal quality.

It is desirable for electrode-electrolyte materials to be electrochemically reversible and stable, with minimal fluctuations in potential over time^2,9^. Efforts have been devoted to developing dry electrodes for biosignal monitoring, such as printed Ag/AgCl-based inks on flexible substrates or textiles^4,10^. Work by Xu et al.^11^ and Haddad et al.^12^ have demonstrated the fabrication of Ag/AgCl based e-textiles comparable with Ag/AgCl solid gel electrodes for biosignal monitoring.

Ag/AgCl coated yarn is the key element in developing e-textile electrodes. A wide range of Ag coated yarns made from a variety of core materials, fibre diameter, Ag film thickness, and quality are available on the market and can be used as starting materials for making Ag/AgCl coated yarns. However, formation of uniform, thin, robust, and stable Ag/AgCl coatings on the yarns is challenging due to the sensitivity of the process on multiple factors such as the coating medium, applied current, thickness of the starting Ag coating on the surface, and susceptibility of the coating itself to mechanical abrasion during long-term use. The listed factors have limited the production of high-quality Ag/AgCl coated yarns. Therefore, developing a system with optimized parameters for continuous production of high-quality Ag/AgCl coated yarns is very important. There have been numerous investigations that have studied the formation of AgCl on Ag metal substrates in chloride solutions through both cyclic voltammetry and potentiodynamic polarization^13–16^. The electrochemical tests allow for the interpretation of the formation and growth processes of AgCl nuclei to films on Ag surfaces, as well as from corresponding observations of the layer morphology and microstructure from different operating conditions^13,15^. The formation of AgCl begins with initial AgCl nuclei formation on preferential sites of the Ag surface, or adsorption of chloride ions through a partial charge transfer with the substrate. This is then followed by the nucleation and growth of the three-dimensional AgCl film in patches across the substrate, until a continuous film is formed^13,14,17^. This part of the process is thought to be controlled by either interfacial, or diffusion-controlled growth kinetics^14^.

This work reports a continuous roll-to-roll electrochemical system for the lab scale fabrication of Ag/AgCl-coated multi-filament nylon yarn that has been developed by the authors^12^, electrochemical characterization of the coating process of AgCl on Ag-nylon yarns, design, and performance of embroidered electrodes for ECG measurement, which provide insights into the stability and performance as an electrode material.

## Methods

### Fabrication of Ag/AgCl e-textile yarns—roll-to-roll coating system

**Figure 1:**
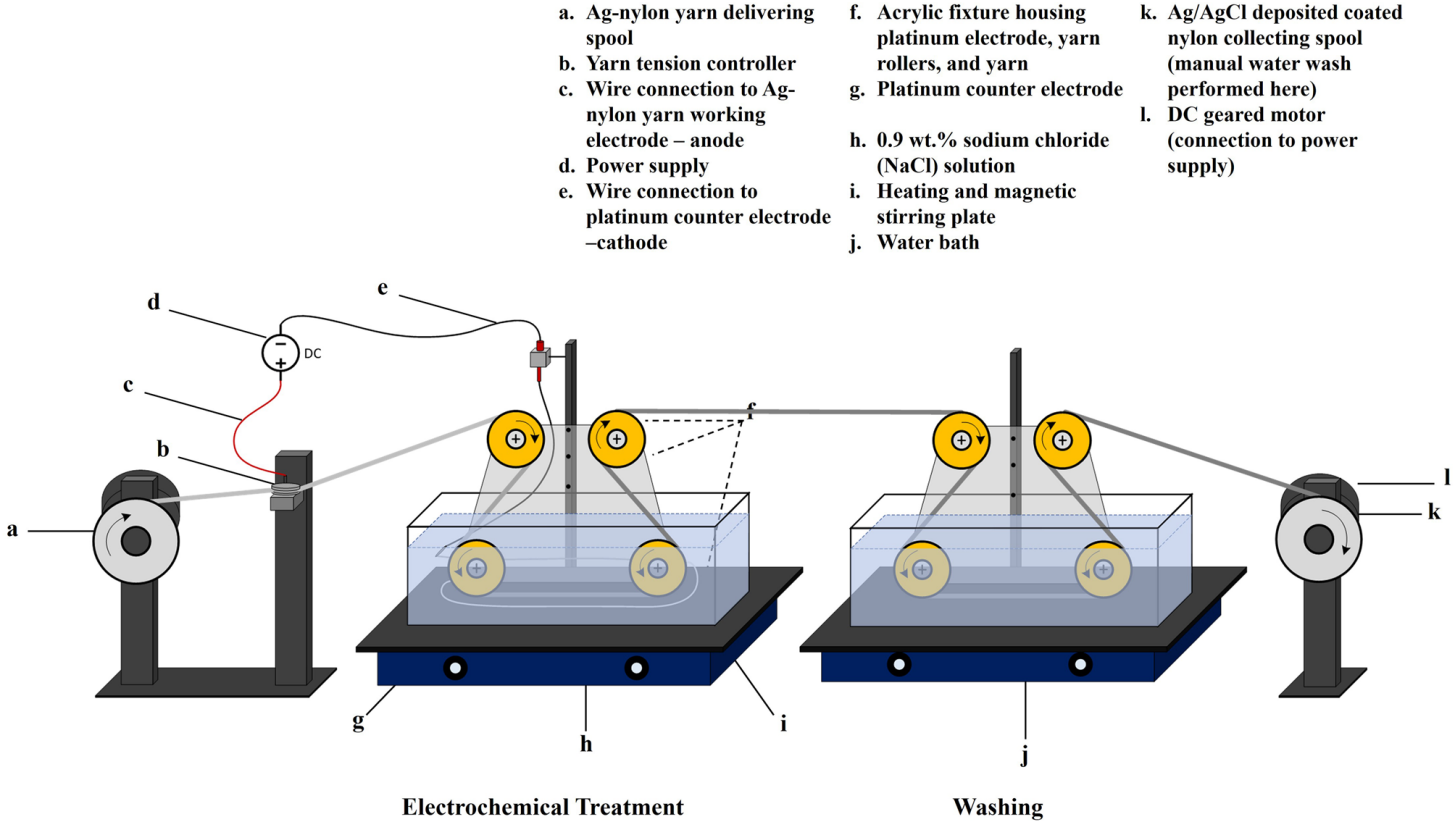
Schematic of roll-to-roll electrochemical coating system.

The method used to fabricate Ag/AgCl yarns is a modified setup from previous work^12^. The setup allows for a higher throughput of Ag/AgCl yarn, as well as modifiable process parameters, which allow for accurate control of coating thicknesses and yarn conductance to meet material performance requirements. Commercial silver (99.9%) coated nylon two-ply yarn (Circuitex™, Noble Biomaterials Inc., USA) with each ply having a 100-denier linear density and 34 filaments, was used as the starting base yarn to be coated. A schematic of the setup is shown in Fig. 1 (additional photograph of setup, Fig. S1). The system comprises multiple components. First a manual tensioner controls the tension of the yarn through the process. A rectangular glass container mounted on top of a magnetic stirrer hot plate is used as the electrochemical reactor. A plexiglass holder consisting of nylon pulleys guides the yarn (working electrode and anode) into the glass reactor to pass through the electrolyte. It is designed in such a way to allow a certain length of the yarn (L) to move horizontally in the electrolyte. Two platinum wires (counter electrode, cathode) with same length of L are horizontally mounted on the holder and stay on both sides and in parallel to the yarn with equal distance from the yarn. Upon exit from the reaction bath, the yarn is directed to the second bath and passes through fresh de-ionized water to wash the yarn and remove any lose particles. At the end, the yarn is wound onto a rotating spool powered by a direct current (DC) geared motor equipped with a speed controller.

The electrochemical process of Ag/AgCl nylon yarn coating was carried out in a 0.9 wt.% NaCl (VWR International, USA) bath. A Keithley 2400 Sourcemeter (Keithley, USA) was used as a current source for the reaction. As the anode, and working electrode, the as obtained Ag-coated yarn passed through a fixture comprised of metal screws/washers and a tension controller. In the setup, this fixture is connected to the positive terminal of the Sourcemeter. The negative terminal of the Sourcemeter is connected to the platinum wires (counter electrode) that are mounted in parallel along both horizontal parts of the yarn at the bottom of the reaction chamber. When the current source is applied, a redox reaction occurs in which the silver-coated yarn (anode) in the NaCl solution undergoes oxidation, to silver ions (Ag^+^) at the electrode–electrolyte interface (Eq. 1a). The silver ions combine with chloride ions (Cl^−^), depositing on the yarn (Eq. 1b). The reaction time is controlled by the speed of the DC motor that winds the yarn through the reaction bath. At the end of the process, the yarn is removed from the spool and dried for 10 h at 60 °C in an oven further characterizations and use.

1a $${\text{Ag}}\left( {\text{s}} \right) \rightleftharpoons {\text{Ag}}^{ + } + {\text{ e}}^{ - }$$

1b $${\text{Ag}}^{ + } + {\text{ Cl}}^{ - } \rightleftharpoons {\text{AgCl}}\left( {\text{s}} \right)$$

### Characterization of yarns and E-textile electrodes

#### Electrochemical characterization

Cyclic voltammetry (CV), and potentiodynamic polarization (PDP) tests were carried out using a Biologic VMP-300 electrochemical workstation (BioLogic, France) to characterize the formation of AgCl on the Ag-nylon yarn, and to identify appropriate operating range of currents for a stable reaction process. The tests were carried out in the 0.9 wt.% NaCl solution, using a three-electrode cell configuration, with the Ag-coated nylon yarn as the working electrode, a platinum wire as the counter electrode, and a saturated calomel electrode (SCE) as the reference electrode.

The CV scans were performed under both steady-state and dynamic conditions, from − 400 to + 150 mV (vs. ref.) at a scan rate of 100 mV/s for 10 cycles. The reference value for the standard electrode potential of Ag/AgCl vs. SCE is − 0.022 V. The CV scans display the redox reactions in the NaCl solution and were used to qualitatively identify points of nucleation and growth of AgCl.

The PDP tests were performed from − 200 to + 1600 mV (vs. ref.) at a scan rate of 0.5 mV/s. The results of the test are used to identify a suitable and stable operating window for the anodizing process of Ag to AgCl.

#### Materials characterization

The linear resistance of yarn samples was measured using a two-probe multimeter (Tektronix DM2040, USA). The reproducibility of the process and uniformity of the coating was evaluated by measuring the resistances of three separate sets of yarns (each 10 cm long) coated under the same processing parameters.

Sheet resistance of the embroidered e-textile electrodes was measured using a four-point probe setup, consisting of four equally spaced spring-loaded gold tips (20 mm), attached to an adjustable stage to minimize sample damage, and accommodate various sample thicknesses. A source meter (Keithley 2400 Sourcemeter, USA) was used to supply constant current to the two outer tips, and a multimeter (Tektronix DM2040) was used to measure the voltage across the two inner probes. From this configuration, the sheet resistance can be obtained by Eq. (2):

2 $${\text{Rs }} = \, \left( {{\text{kV}}} \right)/{\text{I}},$$

where k is the geometric correction factor for non-ideal samples, with k = 4.532 for a semi-infinite sheet^18^. The surface resistance of the embroidered electrodes was measured in both warp and weft (vertical and horizontal) orientations.

X-ray diffraction (XRD) was carried out using a diffractometer (Rigaku Ultima IV, Japan), with CuKα radiation (1.54 Å), 2θ ranging from 5° to 90°, scan step size of 0.02°, scan speed of 3°/min, to verify the crystal structure of the deposited Ag/AgCl.

Scanning electron microscopy (SEM) (Carl Zeiss NTS Ltd., Germany) was used characterize the surface morphology and thickness of the Ag and Ag/AgCl layers (secondary electron signal, 10 kV accelerating voltage). Energy-dispersive x-ray spectroscopy (EDX) was used to qualitatively verify the presence of Ag and AgCl on the coated yarns. To examine the cross-section of the yarns, specimens were cast in epoxy resin (Epofix Struers, Denmark), cured for 48 h, and polished to obtain a flat surface with roughness of less than 2 µm.

#### Electrode fabrication by embroidery

**Figure 2:**
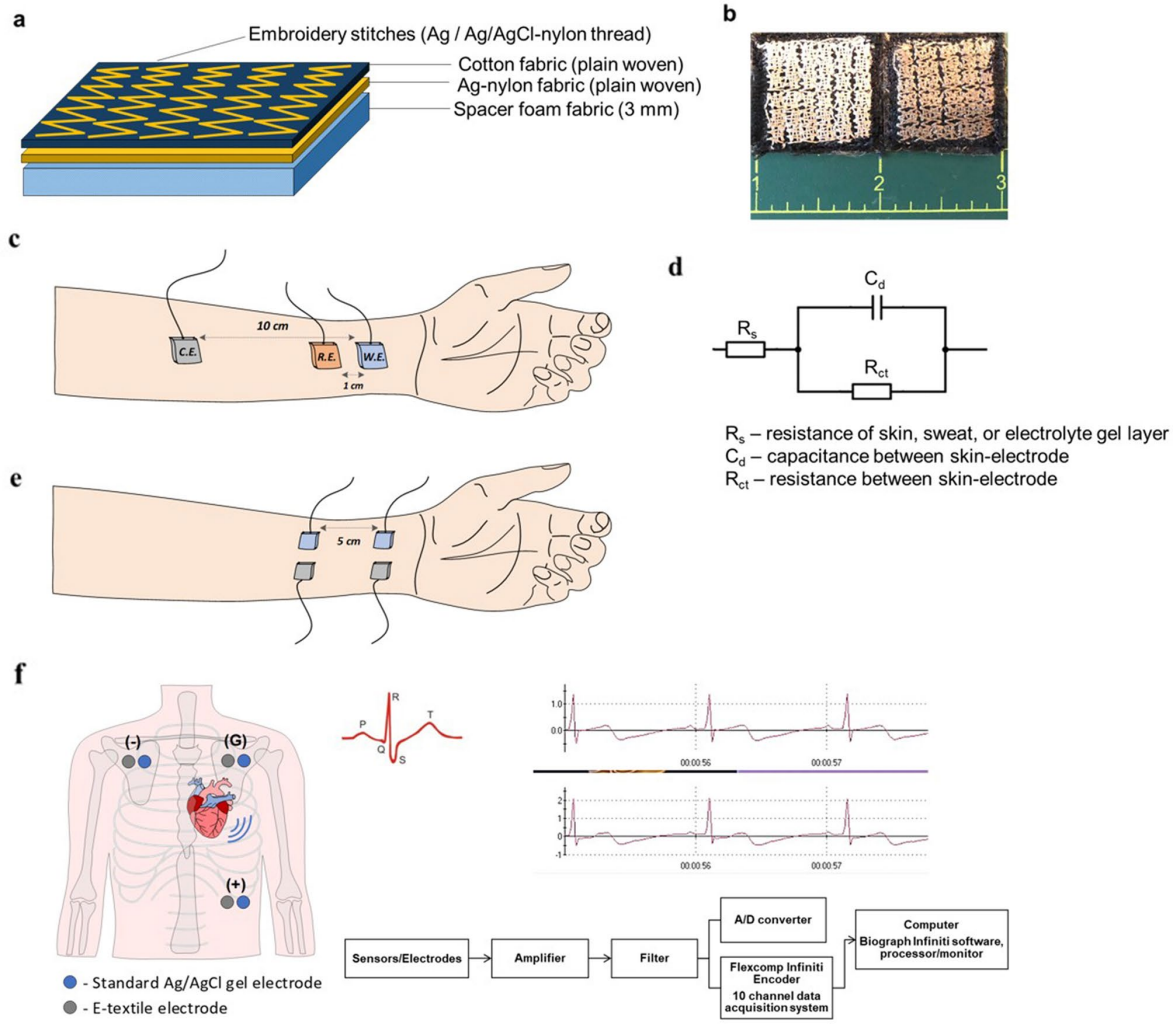
(**a**) Schematic of embroidered electrode structure, (**b**) Embroidered Ag—(left) and Ag/AgCl e-textile electrodes (right), (**c**) Electrode configuration for EIS measurement on planar forearm—CE is Ag or Ag/AgCl e-textile electrode, RE and WE are standard Ag/AgCl gel electrodes, (**d**) Simplified Randle’s Circuit representing skin–electrode impedance, (**e**) Electrode configuration for polarization potential measurement with two sets of electrodes (Ag or Ag/AgCl e-textile and standard Ag/AgCl gel), (**f**) ECG electrode configuration and measurement system schematic.

The e-textile yarns were used to develop new electrodes for biosignal detection. The Ag/AgCl coated yarns were embroidered onto two layers of woven fabric, a 244-gsm cotton woven fabric, and 44-gsm silver-nylon woven fabric (Statex, Germany), using a sewing machine (Baby Lock Aventura Sewing Machine, Suzuki Machinery Co., Ltd., Japan). The silver-coated nylon woven fabric (Statex, Germany) was placed as a backing material for the electrode, to serve as an electrical connection for the electrodes during testing. A zig-zag lockstitch of 5 mm width was patterned into six columns, both the horizontal and vertical directions using one continuous conductive yarn for each electrode (Fig. 2a). The fabric was then stitched to a 3 mm spacer foam. Two sets of 2.5 cm × 2.5 cm electrodes were made from the original Ag and coated Ag/AgCl nylon yarns (Fig. 2b).

#### Electrochemical characterization of e-textile electrodes

The electrochemical characterization was conducted using an electrochemical workstation (BioLogic VMP 300, France). Electrical impedance spectroscopy (EIS) was performed on the left palmar forearm of a 30-year-old female, using a three-electrode measurement setup. The embroidered e-textile electrode was used as the working electrode (WE), while standard Ag/AgCl gel electrodes (UniGel, USA) served as counter and reference electrodes (CE, RE, respectively). The electrodes were positioned following the setup used by Euler et al.^19^ with 10 cm distance between the WE and CE, and 1 cm distance between the WE and RE (Fig. 2c). Using an arm wrap, light pressure (~ 0.5 kPa) was applied to the electrodes to ensure direct contact to skin. The EIS tests performed in potentiostatic mode, in which a sinusoidal voltage of 1 V was applied, using a frequency scan from 10 kHz to 0.1 Hz, with 10 points per decade, sinus amplitude of 10 V_rms_ and three measurements per reading. Analysis was performed by comparing the recorded EIS curves with equivalent circuit models to calculate circuit components. The Simplified Randle’s circuit was selected to represent the skin–electrode interface and used for impedance estimation. The model comprises a resistor Rs in series with a parallel resistor (R_ct_) and capacitor (C_d_) (Fig. 2d). The capacitor represents the electrical charge between the electrode and skin, R_ct_ represents the resistance between skin and electrode during the charge transfer process, and R_s_ represents the total resistance of the body/electrolyte, wires, and electrodes^20,21^.

Electrode polarization occurs due to changes in the charge distribution at the skin–electrode interface, and may cause baseline drift, or DC offset in ECG signals^22^. Therefore, electrodes suitable for ECG or biopotential measurements must be stable enough so that fluctuations do not interfere with the measured signals. This is generally controlled by appropriate material selection (use of Ag/AgCl), or through applying a high pass filter during signal processing^23^. Polarization potential was measured by utilizing the open circuit potential mode. The potential difference was recorded every 0.1 s for 10 min for each e-textile electrode set, placed 5 cm apart on the left planar forearm, and compared with standard gel Ag/AgCl electrodes (Fig. 2e). Mean potential drift for each measurement series (per minute) was calculated according to Eq. (3)^24^

3 $$\frac{{\mathop \sum \nolimits_{i} \left| {x_{i} - x_{i - 1} } \right|}}{i + 1},\;\;{\text{where }}\;{\text{i}}\; \, = \;{ 1}, \ldots {\text{n}}$$

#### Electrocardiogram measurements and analysis measurements

ECG was measured using a clinical 1-lead ECG system (Thought Technology Ltd., Canada), equipped with a data acquisition board and processing software (Biograph Infiniti), at a sampling rate of 2048 samples/s. Signals were simultaneously collected from two channels to compare the performance of the e-textile electrodes with the standard Ag/AgCl gel electrodes. The sets of electrodes were attached side-by-side in a chest placement configuration, following the manufacturer’s guideline (Fig. 2f). It should be noted that the simultaneous recordings do not produce identical ECG signals, since they will pick up slightly different phase and amplitude differences due to relative positioning of the electrodes. The e-textile electrodes were attached to the skin using a snap clip connector compatible with ECG snap leads, and secured with medical tape (3M, USA). Three trials, six-minutes long, were conducted in a stationary seated position for each electrode set (Ag and Ag/AgCl e-textiles). All measurements were performed on one subject (30-year-old, female), with informed consent obtained from the subject. Experimental protocols were approved by the University of British Columbia Clinical Research Ethics Board (UBC CREB: H21-03312) and carried out in accordance with relevant guidelines and regulations.

Methods in the time and frequency domain were used to compare ECG signal quality of the different electrode materials. For time domain comparison, variability (coefficient of variation, % C.V.) in the R-R (or inter-beat-interval IBI) intervals were compared, as well as a visual comparison of the waveforms. In addition, average waveforms were calculated by template matching, from which the Pearson’s correlation coefficient (PCC) was calculated between each e-textile electrode material set and the corresponding standard reference electrode. The template matching method compares similarities in the ECG waveform morphology. A modified model from Orphanidou^24^ was employed, using the entire PQRST waveform^25^, and was carried out in MATLAB (R2021b, The Mathworks Inc., RRID:SCR_001622). PCC results were compared to the metrics for template matching quality indices specified by Orphanidou, with a PCC of greater than or equal to 0.66 deemed as acceptable. Previous work employing this method for commercial Ag-nylon e-textiles reported PCC values of approximately 0.94 (range: 0.91–0.98)^25^. For frequency domain comparison, power spectral density plots were calculated and compared in terms of occupied bandwidth (total, lower, and upper bounds, in Hz), which represents the data region containing 99% of the power spectral density estimate^26^.

### Ethics approval

Biopotential signal monitoring tests were carried out under the approval of the University of British Columbia Clinical Research Ethics Board, UBC CREB: H21-03,312.

## Results and Discussion

### Roll-to-roll system process parameters and yarn electrical properties

The winding speed for the spool collecting the coated yarn was measured from 0.75 to 10 V in 0.5 V increments, corresponding to speeds of 0.022–0.35 cm/s. The corresponding yarn speed as well as the corresponding reaction time were measured, and the results are displayed in Fig. S2a and Table S1. The reaction time was considered as the total time that a certain point of the yarn is immersed in electrolyte between the Pt electrodes in the reaction container. Figure S2b displays a typical plot of resistance of the as-obtained Ag-yarn across a 25 cm segment, measured at every 1 cm. The linear relation of the resistance vs. length of the yarn shows uniform Ag coating with an average resistance of 1.8 Ω/cm. Following previous studies^12^, initial yarn coating was performed at various winding speeds, 0.75–8 V, and at different applied deposition currents, varied from 0.5 to 2.0 mA. This was performed to obtain a general understanding of operating parameters for the modified roll-to-roll system. Like the Ag-coated yarn, the average linear resistance was measured for the Ag/AgCl-coated yarn, and results are plotted in Fig. S2c. Based on the results, operating parameters which result in yarn resistances between 10 and 30 Ω/cm were explored, due to the sharp increase in yarn resistance observed thereafter (for samples measuring > 30 Ω/cm). This is likely due to the full dissolution of the thin Ag-coating on the yarn, measured to be approximately 265 nm from the SEM cross sectional image (Fig. S3).

### Electrochemical characterization

**Figure 3:**
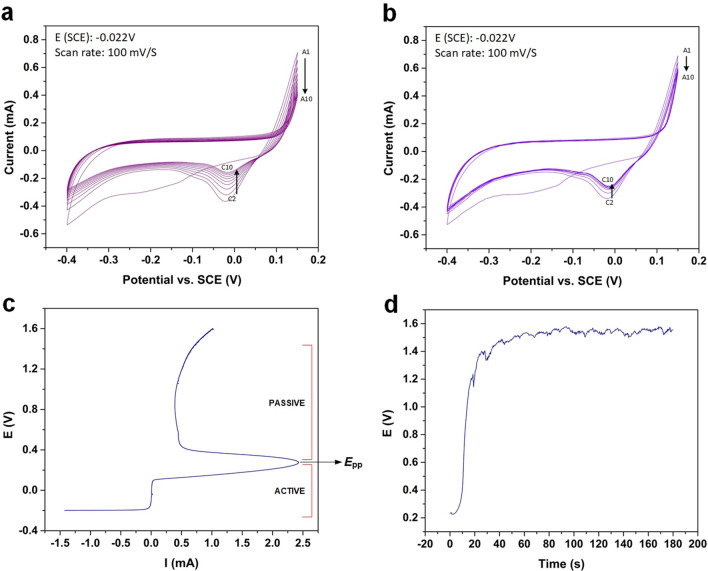
(**a**) CV scans (10 cycles) under static condition in 0.9 wt.% NaCl solution, (**b**) CV scans (10 cycles) under dynamic condition, with yarn winding speed of 0.13 cm/s in 0.9 wt.% NaCl solution, (**c**) PDP measurement for AgCl formation on Ag nylon yarn in 0.9 wt.% NaCl, (**d**) Open circuit potential measurement to assess stability of AgCl coating process over a 3-min period.

The CV tests were used as a tool to understand the redox reaction window of the electrochemical coating process of Ag-nylon yarn in 0.9 wt.% NaCl solution. Figure 3a displays recorded results of 10 CV-cycles (0.5–0.2 V) carried out for the process under steady-state conditions, in which the yarn was kept stationary (no yarn movement) in the electrolyte solution. As can be seen, an anodic peak was observed at scan potential of ~ 148 mV. Prior to this, the forward scans show nearly zero current, demonstrating the inert behavior of Ag under the conditions. The intensity of the anodic peak decreases upon subsequent scans, owing to the stripping of the Ag layer from the yarn surface^15^ (Eq. 4a). Furthermore, a broad shift is observed from the anodic to cathodic peak in the reverse scan and demonstrates a multi-step process occurring in the anodic region, described by Eq. (4b), representing the formation of silver chloride nuclei.

In the cathodic scan, the peak potential shows a positive shift, increasing from − 21 to − 15 mV. This positive shift may indicate a more efficient electron transfer process due to the formation of conductive Ag and Ag adatoms on the surface of the yarn. However, over the increasing scans, the decreased peak area demonstrates a lower reaction rate with increasing scans. As this test was carried out under stationary conditions (yarn in solution), this is indicative of the conversion of Ag to Ag/AgCl being limited by the available Ag coating on the nylon yarn surface. The reactions occurring in the cathodic scan can be explained by work from Birss and Smith^13^, who studied the formation of AgCl through surface enhanced Raman scattering effect (SERS), to involve the deposition of silver adatoms when AgCl nuclei reduce (Eqs. 5a, 5b). The adatoms are active forms of silver that may enable the formation of the AgCl film in subsequent anodic cycles.

1. **Anodic peaks** (multi-step process)
  4a $${\text{Ag}}_{( {\text{s}})} \rightleftharpoons {\text{Ag}}^{+} \, + e^{-}$$
  4b $${\text{Ag}}_{( {\text{s}} )} \, + {\text{ Cl}}^- \rightleftharpoons {\text{AgCl}}_{( {\text{s}} )} \, + {\text{ e}}^-$$
2. **Large cathodic peaks** (Ag adatoms)
  5a $${\text{AgCl}}_{( {\text{s}} )} \, + {\text{ e}}^- \rightleftharpoons {\text{Ag}}_{( {\text{s}} )} \, + {\text{ Cl}}^-$$
  5b $${\text{Ag}}^{+} \, + {\text{ e}}^{-} \rightleftharpoons {\text{Ag}}_{( {\text{s}} )}$$

The CV tests were also performed under dynamic condition when the Ag-coated yarn was moving through the solution bath at a speed of 0.13 cm/s (4 V motor speed). The recorded results display similar behaviour to the steady-state condition, indicating no significant difference between the reactions occurring under the two conditions (Fig. 3b). It should be noted that smaller shifts were observed in the anodic and cathodic peaks with increasing scans, owing to continuous movement of the yarn, working electrode, and continuous exposure of fresh yarn to the electrolyte in the reaction bath.

Potentiodynamic polarization (PDP) was performed to determine the suitable operating range of applied currents for AgCl formation. The test follows the process parameters specified by Pargar et al.^15^ Shown in Fig. 3c, the initial potential increases with current (active corrosion), until it reaches the primary passive potential, E_pp_, at approximately 0.27 V, and up to 2.4 mA. Thereafter, current and corrosion rate decrease (passive region), in which an external applied potential is needed to activate the process. This can be interpreted as Ag beginning to passivate after 0.27 V, forming AgO. Therefore, as only Ag dissolution is desired to subsequently form AgCl, it is best to operate the process at potentials lower than 0.27 V, or current lower than 2.4 mA. Based on the above observation, the applied current of 2 mA (or 2.3 mA/cm^2^) was selected for Ag/AgCl yarn fabrication.

To study the stability of the roll-to-roll electrochemical coating process, the potential (using open circuit voltage measurement) was measured during the reaction process, by attaching the probes of the electrochemical workstation (BioLogic VMP300, France) to the working electrode (Ag yarn), counter electrode (platinum wire), with a SCE as a reference electrode. The measurement was performed for 3 min at a constant applied current of 2 mA, while the Ag-coated nylon yarn was moving through the reaction bath at a rate of 0.13 cm/s (4 V motor speed). The recorded measurement results are plotted in Fig. 3d, showing that the reaction process is stable. It takes approximately 30 s to reach a steady potential, as yarn resistance increases with the AgCl film formation.

### Yarn electrical and material characterization

**Figure 4:**
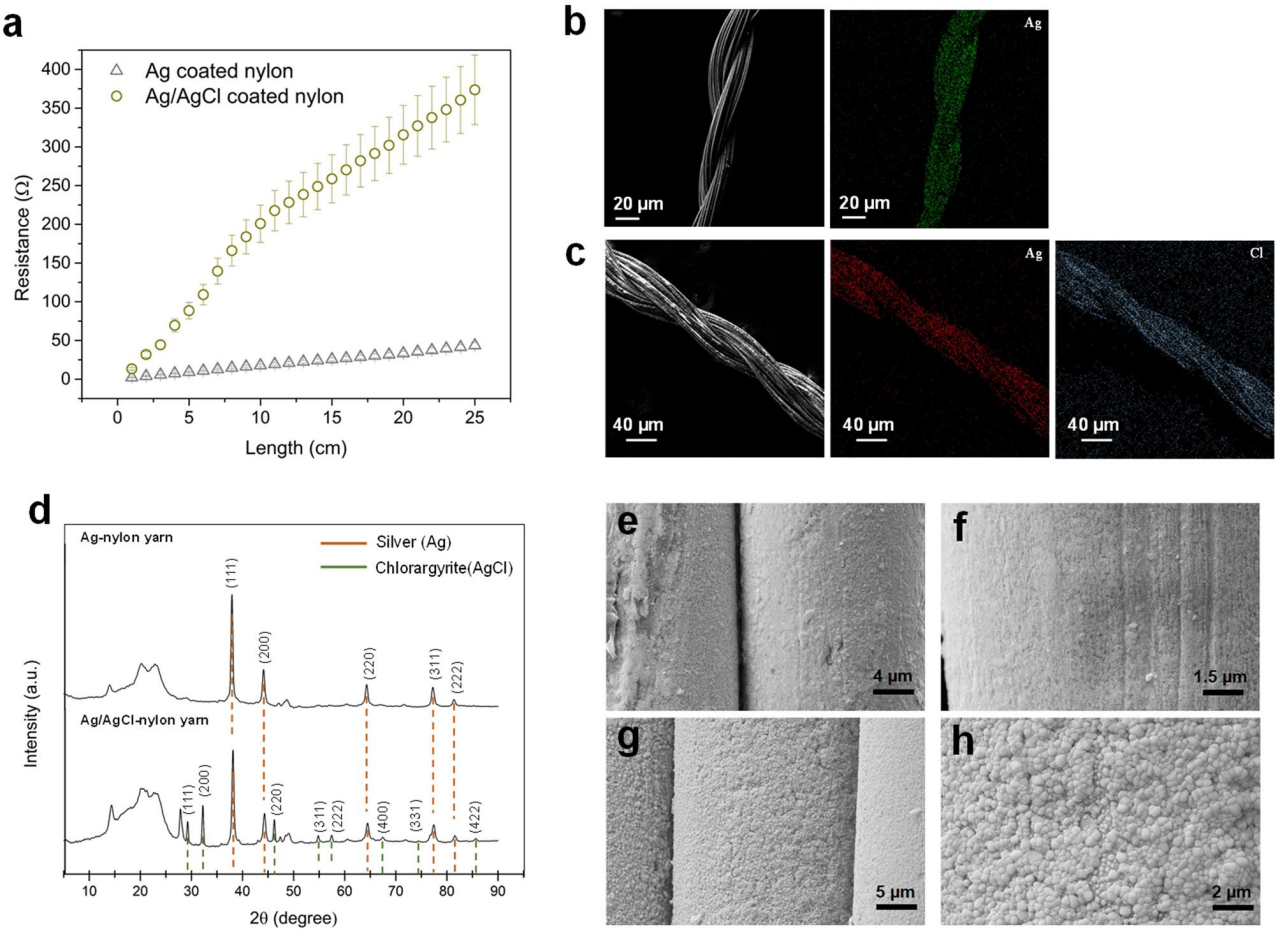
(**a**) Resistance of Ag-coated nylon (original), and Ag/AgCl coated nylon across 25 cm yarn length, (**b**) Ag-coated nylon yarn, with EDX map of Ag, (**c**) Ag/AgCl-coated nylon yarn, with EDX map of Ag and Cl, (**d**) XRD patterns for yarn surfaces of Ag-coated nylon yarn, and Ag/AgCl-coated nylon yarns, with annotated peaks corresponding to silver and silver chloride (**e**) SEM micrographs of Ag-coated nylon yarn surfaces at 3.5k ×, (**f**) 8.5k × magnifications, (**g**) SEM micrographs of Ag/AgCl-coated nylon yarn surfaces (2.0 mA applied current) at 4k ×, and (**h**) 12k × magnifications.

The measured resistance as a function of yarn length for the original Ag-coated yarn, and Ag/AgCl-coated yarn, processed at 2 mA (0.13 cm/s, 4 V motor speed) are shown in Fig. 4a. The average linear resistance of the original Ag yarn is 1.8 ± 0.2 Ω/cm (n = 25), while the coated Ag/AgCl yarn is 14.7 ± 4.7 Ω/cm (n = 50).

Further qualitative material characterization was performed on the Ag-coated and Ag/AgCl-coated nylon yarns by SEM–EDX (Fig. 4b, c) and XRD (Fig. 4d) analysis. Figure 4b, c show the EDX maps for the Ag and Cl distribution on the yarn surfaces (spectrum analyses for Ag and Ag/AgCl yarns included in Fig. S4a, b). Both Ag and Cl were similarly observed all over the yarns. This confirms the uniform distribution of Ag on the original yarns, and uniform reaction process of Ag/AgCl coating on the surface of the processed yarns.

The SEM micrographs of the surfaces (Fig. 4e, h) show differences in the morphology of the Ag and Ag/AgCl yarns. The Ag yarns appear to have a smoother and continuous surface (Fig. 4e, f), whereas the Ag/AgCl coated yarns show a granular surface with varied grain size (average ~ 220 ± 30 nm) and stacked layers of grains (Fig. 4g, h). In addition, at higher magnifications, pores are visible on the outer layers (Fig. 4h), characterized as microchannels, that facilitate the ionic transport through the film^14–16^. According to characterization work by Ha et al.^14^, initial AgCl layer growth begins in a non-continuous manner, in which Ag dissolution kinetics can be described as activation control. With a thin layer formed, and patches of AgCl expanding laterally across the substrate, dissolution kinetics become mixed control, with ohmic overpotential through the AgCl layer, and activation overpotential in Ag substrate regions. Ionic transport occurs through the newly formed film through spaces in between the AgCl grains. As a thicker film forms, ionic transport occurs through micro-channels through the grain, upon which any further ionic dissolution occurs as ohmic overpotential through the AgCl layer.

As observed from the XRD analysis spectrum, AgCl forms in a polycrystalline structure, with clear peaks associated with (220), and (111) crystal planes, which reflect the angular and spherical grains as described by Zhang et al.^16^. The grains are clearly visible on the microstructure surface of the coated yarns, shown in Fig. 4h. The peaks observed below 25° could not be identified, they likely reflect the exposed nylon 6,6 core of the yarn specimen that was cut and attached to a thin polymer sheet placed on the sample holder.

### Electrode characterization

#### Surface resistance

**Figure 5:**
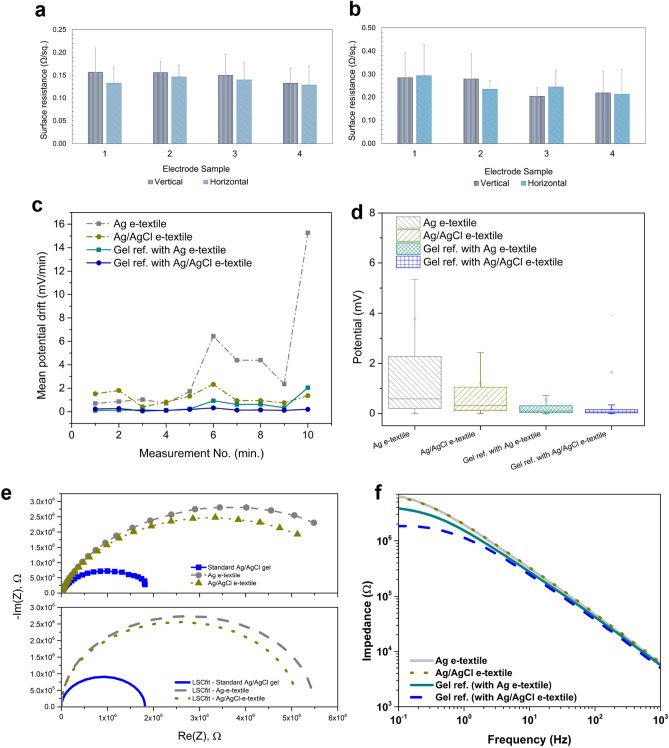
(**a**) Results of surface resistance measurements Ag-nylon embroidered electrodes, and (**b**) Ag/AgCl-nylon embroidered electrodes, (**c**) Mean polarization potential across 10-min measurement period, (**d**) Boxplot displaying range/spread of potentials measured across 10-min period, (**e**) Example Nyquist plot with measured and fitted data, (**f**) Bode plot displaying impedance vs. frequency for e-textile and standard gel Ag/AgCl electrodes.

The surface resistances of a set of four embroidered electrodes were measured and summarized (Fig. 5a, b). It should be noted that the error for the measurements is high due to the uneven contact points between the probe and the embroidered surface.

The average surface resistance measured in the vertical and horizontal directions for the four Ag electrodes are measured as 0.15 ± 0.04 Ω/sq. and 0.14 ± 0.03 Ω/sq., respectively. There was no significant difference between the surface resistance in the two measurement directions for the Ag e-textile electrodes (p-value = 0.33). The average surface resistance for the four embroidered Ag/AgCl electrodes in the vertical and horizontal directions were measured to be 0.25 ± 0.12 Ω/sq. and 0.25 ± 0.10 Ω/sq., respectively. Again, no significant differences between measured surface resistance for the two directions were observed (two measurement directions for Ag/AgCl e-textile electrodes: p-value = 0.49).

The polarization potential was measured for a period of 10-min for the electrodes made of the Ag-coated and Ag/AgCl coated yarns, as well as the commercially available standard sintered electrodes (with gel electrolyte). The drift for the electrodes were then calculated (measurement taken every 0.1 s, averaged per minute) based on Eq. (3), and summarized in Fig. 5c, d. Polarization potential drift is a measure of electrode stability over time. It is important that electrodes have minimal polarization potential fluctuations to minimize interference with biosignals being collected. As observed in Fig. 5c, the Ag e-textile electrodes show higher variation in mean polarization potential, notably after 5 min of measurement (average drift 3.79 mV/min, between 0.71 and 6.44 mV/min), and a higher overall average compared with the Ag/AgCl e-textiles (average drift 1.22 mV/min, between 0.41 and 2.33 mV/min), and reference gel electrodes (average 0.18 mV/min, between 0.06 and 0.32 mV/min). It should be noted that dry textile electrodes have been compared with commercially available standard electrodes that contain gel electrolyte. The gel fills the gaps between the electrode and skin, which minimizes drift.

To estimate the potential effect of drift on the measured signal, a calculation method utilized by Rattfalt et al.^27^ is followed. The QRS wave of ECG can be considered: assuming an amplitude of 1 mV and 80 ms duration,^28^ this yields a rate of 12.5 mV/s. Scaling the results for the average drift of e-textiles yields 0.06 mV/s, and 0.02 mV/s for the Ag and Ag/AgCl e-textile electrodes, respectively. This represents a factor of 103 difference, which indicates that the e-textiles would be unlikely to cause significant drift on the ECG signal. In comparison, work by Rattfalt et al.^23^ reported the mean polarization potential drift for textile electrodes (stainless steel and polyester blend) to range from 2 mV/min up to 16 mV/min.

Overall, the low potential measured demonstrate the adequate stability of Ag/AgCl e-textile electrode materials for ECG and heart rate measurement. To further reduce the potential drift, a denser embroidered structure of Ag/AgCl yarn can be considered.

#### Skin–electrode impedance

The skin–electrode impedances of the e-textile electrodes were measured and compared to the standard Ag/AgCl gel electrodes. Estimates for the components of the skin–electrode impedance model were made using the Simplified Randle’s circuit (Fig. 2d), and R_s_, R_ct_, and C_d_ were calculated in MATLAB (R2022a, Mathworks) using the least squares nonlinear curve fitting method. Example results are plotted in Fig. 5e, f and summarized in Table 1.

**Table 1:** Summary of average impedance results at 1 Hz, 10 Hz, 145 Hz, and estimates of circuit model parameters for electrode materials investigated.

	Z @ 1 Hz (MΩ)	Z @ 10 Hz (MΩ)	Z @ 145 Hz (MΩ)	R_s_ (MΩ)	R_ct_ (MΩ)	C_d_ (F)
Ag	1.890	0.300	0.034	6.33 × 10^–4^	5.62	2.41 × 10^–8^
Ag/AgCl	1.892	0.306	0.035	6.50 × 10^–4^	5.35	2.36 × 10^–8^
Ref. Gel	1.288	0.241	0.029	6.37 × 10^–4^	3.53	2.52 × 10^–8^

Model parameters were estimated from the following equations:

5a $$Z{ } = R_{s} + { }\frac{{R_{d} }}{{\left( {1 + X_{d}^{2} } \right)}}{ } - j\frac{{R_{d} X_{d} }}{{\left( {1 + X_{d}^{2} } \right)}}{ }$$

5b $${\text{where}}\;X_{{\text{d}}} \, = \,R_{{\text{d}}} \cdot C_{{\text{d}}} \cdot \omega , \, \omega \, = \,{2}\pi {\text{f}}$$

6a $$\left| {\text{Z}} \right|^{2} { } = {\text{ A}}^{2} + {\text{B}}^{2} { ,}$$

where the real and imaginary components of |*Z*| as A and B as shown:

6b $$A{ } = R_{s} + { }\frac{{R_{d} }}{{\left( {1 + X_{d}^{2} } \right)}}$$

6c $$B = - { }\frac{{R_{d} X_{d} }}{{\left( {1 + X_{d}^{2} } \right)}}{ }$$

Overall, both Ag and Ag/AgCl e-textile electrode showed nearly identical impedance across the range of 0.1–10 kHz (Fig. 5f), but higher than that for the standard gel reference electrodes. In general, impedance between 0.01 and 5 MΩ in the 5–100 Hz range is deemed acceptable for ECG e-textiles^29–32^. The impedance of the e-textile electrodes is within this range, lower than 1.9 MΩ, as summarized in Table 1. The results for the circuit elements for the sets of electrode materials are all within the same order of magnitude and range (Table 1). From the Nyquist plots (Fig. 5e), it is observed that the ohmic resistance contribution is negligible, as the intercept at the highest frequency lies close to the origin. This represents R_s,_ total resistance of the body/electrolyte, wires, and electrode. Furthermore, the plots (Fig. 5e) show visible differences in the charge transfer resistance between the electrode materials (distance between the two axis intercepts). The standard gel electrodes have the lowest, followed by the Ag/AgCl e-textiles, and Ag-e-textiles. This is expected, as the gel electrolyte and AgCl layer on the standard electrodes, and the coated yarns serve to reduce the resistance of ion to electron charge transfer. Both sets of e-textile electrodes have similar estimated values for the components of the circuit model, with the Ag/AgCl e-textiles having a slightly lower resistance and capacitance for the skin–electrode interface. This is understandable as AgCl acts as a bridge for ion to electron transfer from the skin to electrode. The standard gel reference electrodes have slightly higher R_s_ and C_d_ values, likely due to the gel electrolyte layer present on the surface of the electrode, which results in an increased resistance from the skin tissue and electrolyte as R_s_, as well as a capacitive effect, C_d_, between the layers. As expected, the resistance between skin–electrode, R_ct_ for the gel electrode is lower than the e-textile electrodes due to high contact surface with the skin, and high ionic conductivity of the gel, which helps to facilitate ion to electron transfer.

### Electrocardiogram measurements from E-textile electrodes—signal quality evaluation

**Figure 6:**
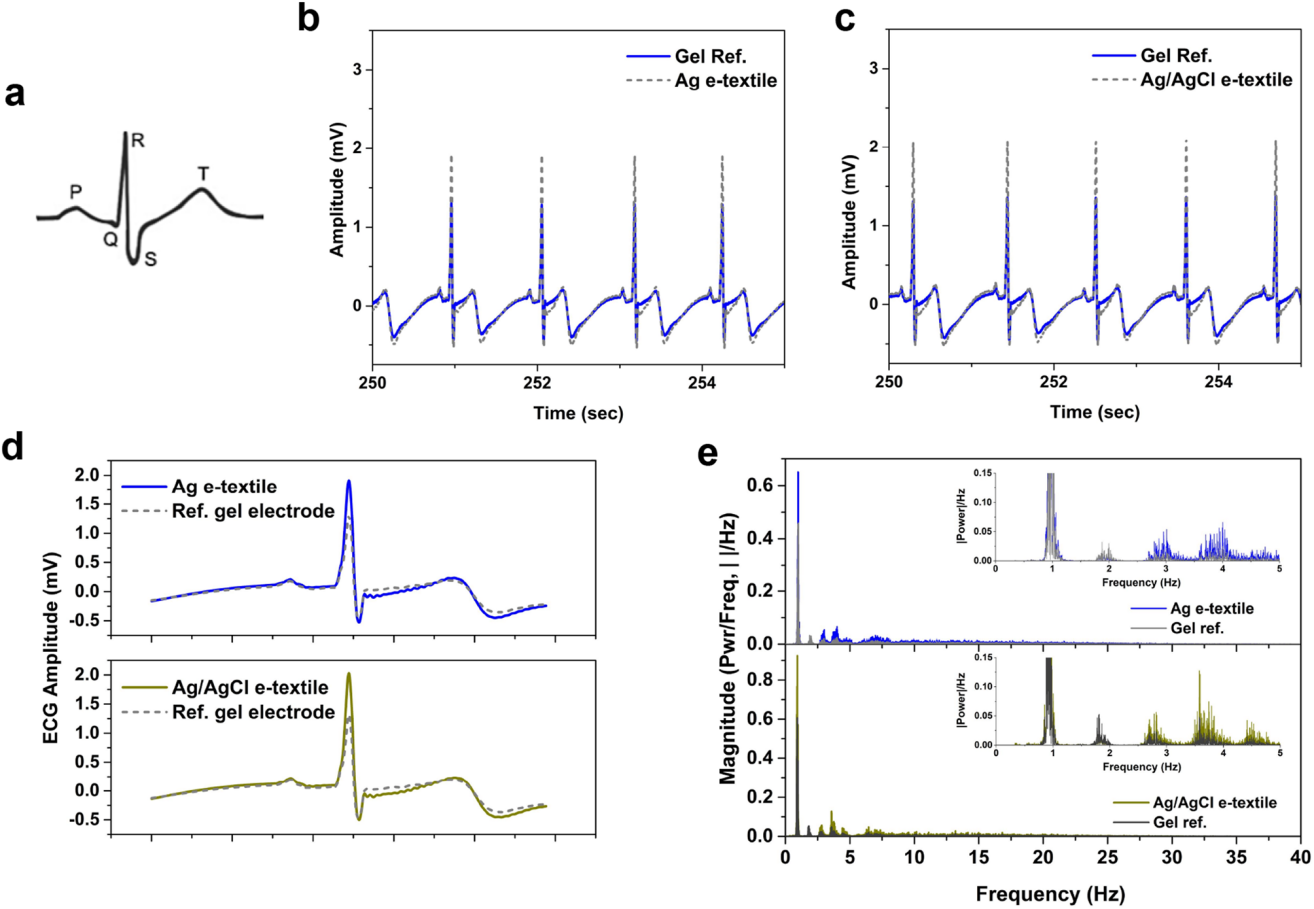
(**a**) Characteristic ECG waveform, (**b**) Sample visual comparison of ECG recording collected for (**b**) Ag- and (**c**) Ag/AgCl e-textiles simultaneously with standard gel Ag/AgCl electrodes, (**d**) Example average ECG waveform (PQRST) for Ag and Ag/AgCl coated nylon e-textiles, (**e**) Example spectral plots for Ag coated nylon, and Ag/AgCl coated nylon embroidered e-textile electrodes compared with standard Ag/AgCl gel electrodes.

Figure 6a shows a characteristic ECG waveform, which represents atrial (P wave), and ventricular (QRS complex) depolarization, followed by the ventricular repolarization (T wave) of the heart. Methods in the time and frequency domain have been applied to compare signal quality between standard Ag/AgCl gel electrodes and the embroidered Ag and Ag/AgCl coated e-textile electrodes.

Initial visual comparison of ECG collected for each e-textile electrode group and corresponding the standard gel electrodes (simultaneous measurement side-by-side) show similar signals, with the embroidered electrodes showing higher R-peak amplitudes, and lower S-waves (Fig. 6b, c). However, the amplitude differences do not impact the heart rates (HR) calculated from the raw signal. The differences are thought to be due to the closer proximity and placement of the textile electrodes to the heart centre compared with the gel electrodes in the simultaneous recording configuration. Another possible reason is due to the higher surface area of the textile electrode compared with the standard gel electrode, which could contribute to better contact between the skin and electrode. Measurements to compare differences in electrode placement on signal R-peak amplitude are summarized in Figs. S5, S6. Corresponding average HRs calculated from the inter-beat intervals (time difference between R-R peaks across a measurement period), standard deviations (SD) and coefficient of variation (CV, %) for all sample groups of the five trials performed demonstrate nearly identical HR results (Table S2). This indicates that embroidered electrodes are excellent alternatives for collecting ECG signals.

The average ECG waveform for the e-textile electrodes were obtained and compared with the respective standard reference (gel) electrode using MATLAB (R2022a, Mathworks, Inc., RRID:SCR_001622). Typical results for the Ag and Ag/AgCl textile electrodes are shown in Fig. 6d. The Pearson’s correlation coefficient (PCC) comparing the simultaneous Ag/AgCl standard gel electrode and e-textile electrode signals were calculated for each trial and summarized in Table S3. Results for both sets of e-textile electrodes have an average PCC value of 0.98, which exceeds the acceptable range defined by Orphanidou, with PCC ≥ 0.66^24^. It falls in the higher end of the acceptable PCC range from previous work comparing Ag-nylon e-textile electrodes, with calculated values of approximately 0.94 (range: 0.91–0.98)^25^.

Analysis of the ECG signals in the frequency domain included the calculation of power spectral density, and occupied bandwidth, representing the data region containing 99% of the total power, with example results displayed in Table 2. The corresponding power spectral density plots for the embroidered e-textile electrodes compared to standard gel electrodes (Fig. 6e) show the higher power magnitude in the low frequency range (1–10 Hz) for the e-textile electrodes, which reflect noise observed in the raw signal (time domain). This observation also corresponds to the occupied bandwidth results that show 99% of the total power to be in a slightly smaller range for the e-textile electrodes (34.56 and 33.86 for Ag and Ag/AgCl e-textiles, respectively), compared with the Ag/AgCl gel reference electrodes (approximately 38.8 for both sets tested simultaneously with e-textile electrodes). Despite these differences, the results for the textile electrodes are within close range to the standard gel electrodes, and demonstrate acceptable ECG signal quality based on the comparison methods employed in this study. Coupled with electrode characterization results, the Ag/AgCl are shown to demonstrate higher stability over a longer recording period, and good ECG signal quality compared to the Ag e-textile electrodes.

**Table 2:** Example results for occupied bandwidth calculations for e-textile and gel electrode ECG signals.

	Occupied bandwidth	Lower bound (Hz)	Upper bound (Hz)
Gel Ref.	36.80	0.87	37.67
Ag e-textile	34.56	0.88	34.49

	Occupied bandwidth	Lower bound (Hz)	Upper bound (Hz)
Gel Ref.	36.52	0.82	37.34
Ag/AgCl e-textile	33.86	0.81	34.67

## Conclusions

This work has demonstrated an electrochemical roll-to-roll system for fabrication of Ag/AgCl coated nylon yarns, which offers high material throughput, and tunable process parameters to obtain high performance materials for fabrication of textile-based electrodes and sensors for biosignal monitoring. The AgCl film growth and morphology were investigated through electrochemical methods and observation by scanning electron microscopy. The appropriate operating current range specific to the yarn studied in this work has been identified as 0.1–2.4 mA. The selected operating parameters of 2 mA applied current, and winding speed of 0.13 cm/s were observed to be stable and reproducible over multiple coating periods, producing Ag/AgCl-nylon yarns with an average resistance of 14.7 ± 4.7 Ω/cm. The electrodes fabricated by embroidery using the coated yarns demonstrated high stability, with low average polarization potential (1.22 mV/min) compared with electrodes embroidered from Ag-nylon yarns (3.79 mV/min), and low impedance (below 2 MΩ between 0.1 and 150 Hz). This demonstrates the improved effect of the metallic salt layer at the skin–electrode interface. The e-textile electrodes were used for ECG signal collection and exceeded the comparative measures for acceptable and good signal quality. Ag/AgCl based e-textiles are flexible, comfortable, and breathable alternatives to standard rigid gel electrodes and have the potential to be integrated unobtrusively into wearable systems for health monitoring applications. Further research for developing Ag/AgCl e-textiles is necessary to address improvements to durability, washability, and long-term performance.

### Supplementary Information

Supplementary Information.

## Data Availability

The raw data and code used for data analysis in this study are available from the corresponding authors upon request.
