# Supplementary material for: Roll-to-roll fabrication of silver/silver chloride coated yarns for dry electrodes and applications in biosignal monitoring
Source: Sci Rep. 2023 Dec 1;13:21182. doi: 10.1038/s41598-023-48245-8 (PMC10692073; doi:10.1038/s41598-023-48245-8)
Supplement: Supplementary file 1 — Supplementary Information. [file 41598_2023_48245_MOESM1_ESM.docx]

**Supporting Information:**

**Roll-to-Roll Fabrication of Silver/Silver Chloride Coated Yarns for Dry Electrodes and Applications in Biosignal Monitoring**

Katherine Le,^a,*^ Saeid Soltanian,^b,^ Harishkumar Narayana, ^b,^ Amir Servati,^b^ Peyman Servati ^b,*^, and Frank Ko^a,^[[1]](#footnote-2)^*^

^a^ Materials Engineering Department, University of British Columbia, Vancouver, BC V6T 1Z4, Canada

^b^ Electrical and Computer Engineering Department, University of British Columbia, Vancouver, BC V6T 1Z4, Canada


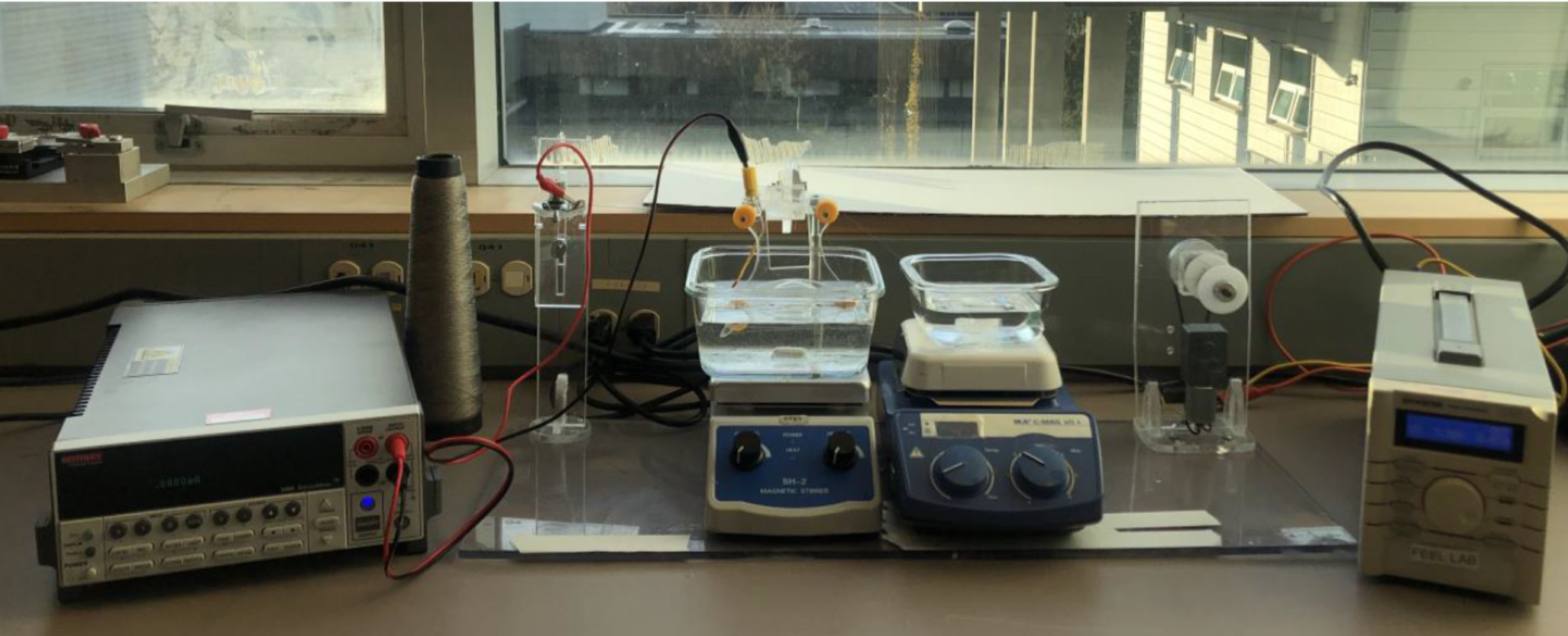


Figure S1 Photograph of continuous roll-to-roll fabrication system.


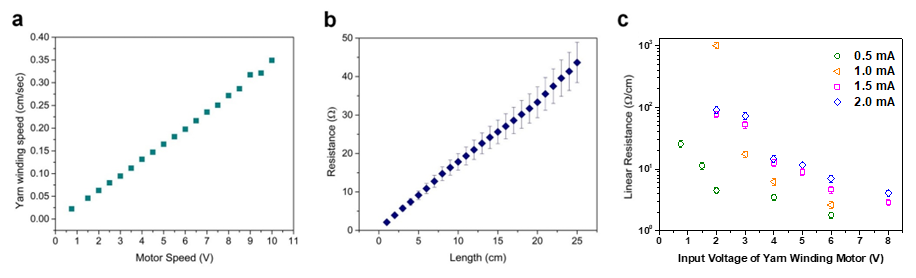


**Figure S2.** a) Yarn winding speed (cm/s) calculated from motor speed (V), b) Linear resistance of the original Ag coated nylon yarn measured across a 25cm segment. Average resistance 1.8 ±0.2 Ω/cm, c) Linear resistance was measured for the Ag/AgCl coated nylon yarns at different applied currents between 0.5 mA to 2.0 mA, and input voltages of yarn winding motor (0.022 cm/s to 0.272 cm/s).

**
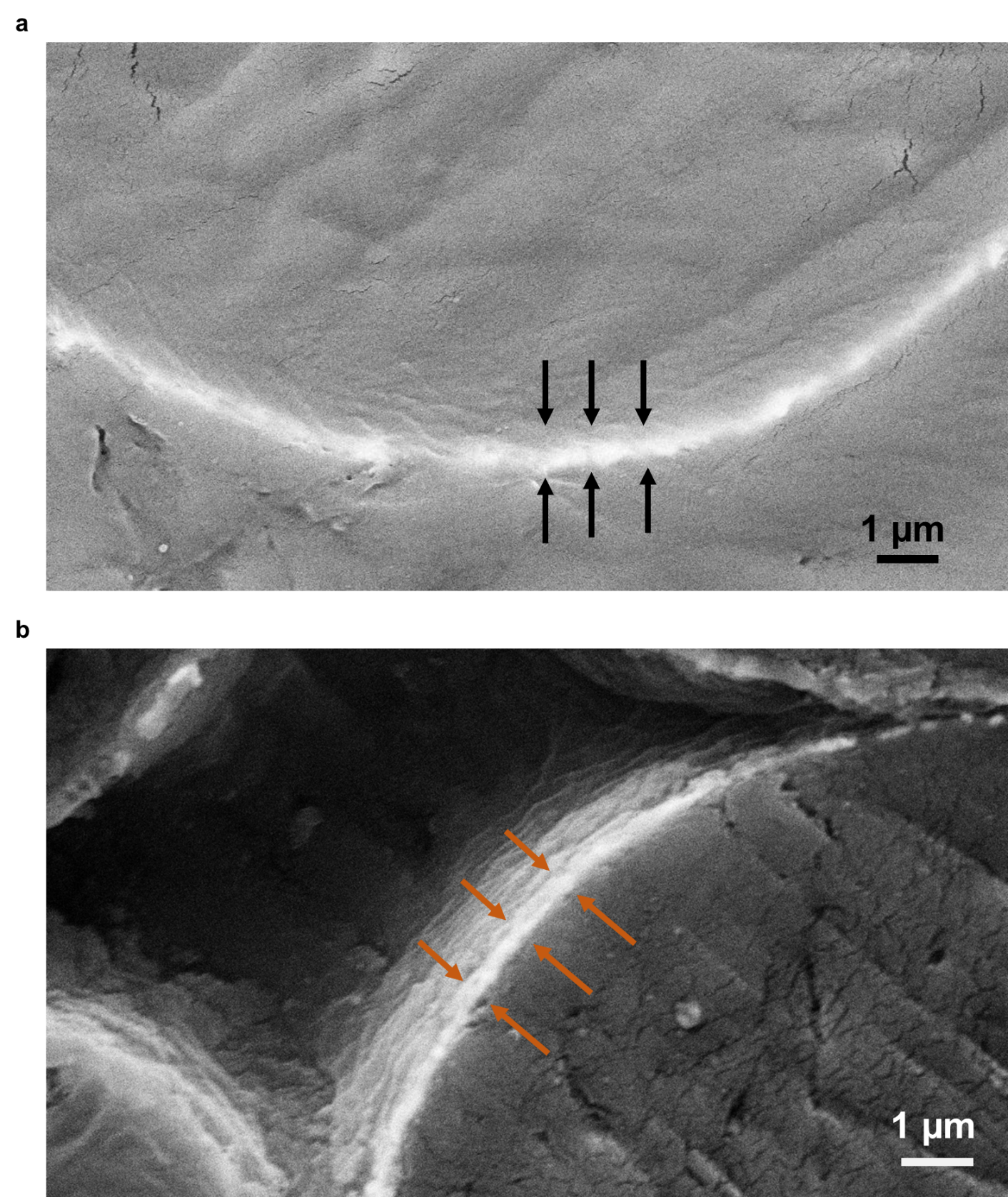
**

**Figure S3.** (a) SEM micrograph of the lower portion of Ag-nylon fibre cross-section, showing Ag coating thickness (bright/white region around perimeter of fiber) (Magnification: 7.5K x).

(b) SEM micrograph of the lower portion of Ag/AgCl-nylon fibre cross-section, showing Ag/AgCl coating thickness, Ag and Ag/AgCl not discernable (bright/white region around fibre edges) (Magnification: 8.5K x)


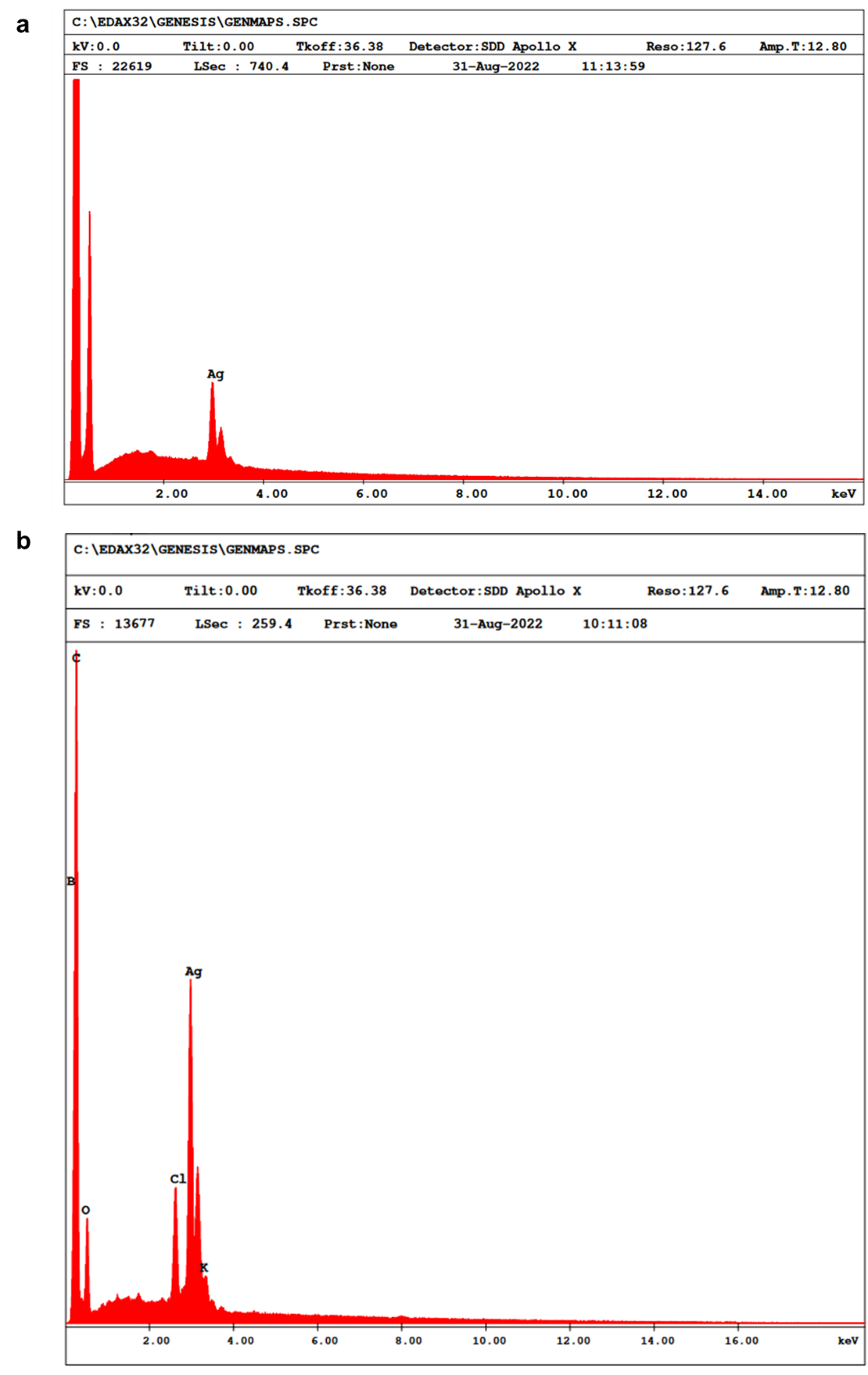


Figure S4. EDX spectrum of a. Ag and b. Ag/AgCl yarns


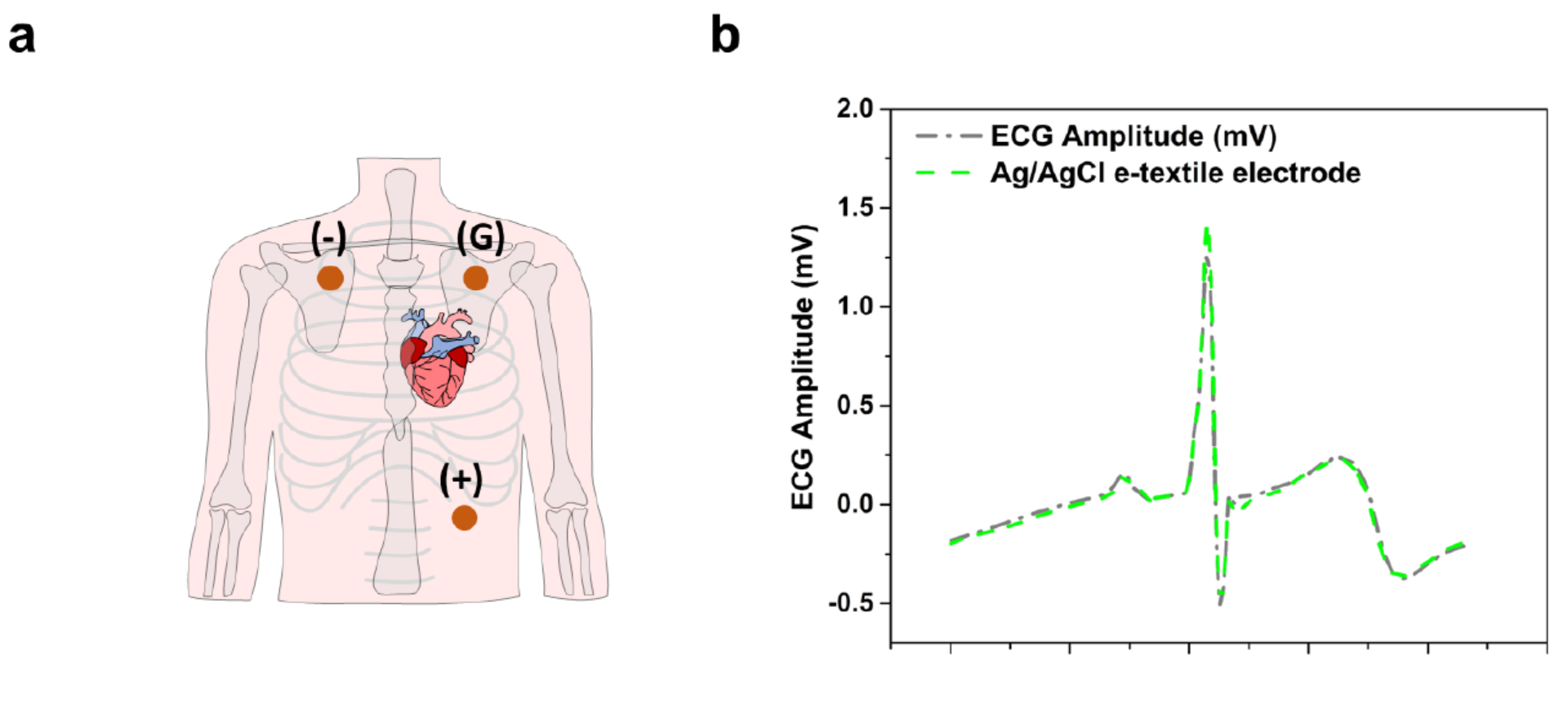


Figure S5. (a) Electrode placement for ECG recording (separate measurements made for standard electrode, and and e-textile set) (b) Average ECG waveform from standard Ag/AgCl gel electrode (R-peak amplitude: 1.27 mV), and Ag/AgCl e-textile electrode (R-peak amplitude: 1.42 mV)


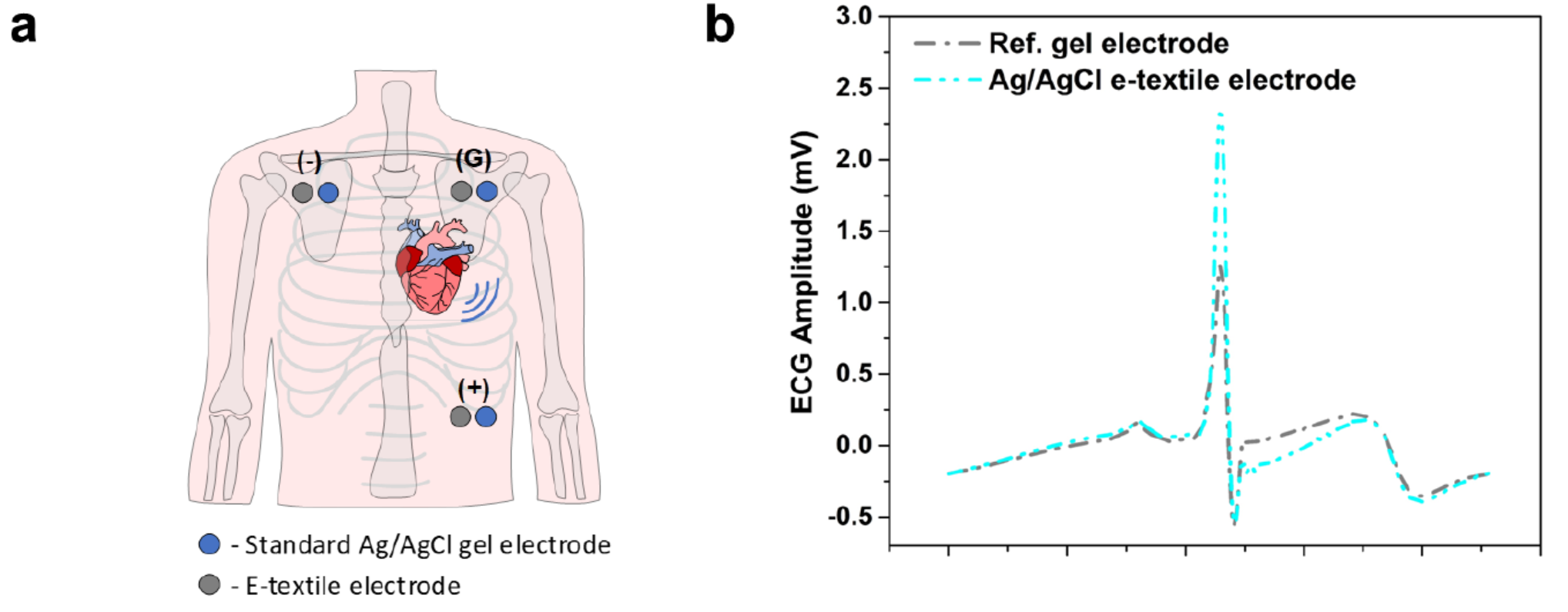


**Figure S6.** (a) Electrode placement for simultaneous ECG recording with standard electrode, and e-textile electrode sets (b) Average ECG waveform from standard Ag/AgCl gel electrode (R-peak amplitude: 1.27 mV), and Ag/AgCl e-textile electrode (R-peak amplitude: 2.36 mV)

*The results show that for ECG recordings made at the same chest location, the R-peak amplitude between the two material sets (gel and textile) are relatively similar. With the simultaneous recording configuration, higher R-peak amplitude is measured for the e-textile electrode set that is placed closer to the heart.*

Table S1. Reaction time of 10 cm yarn segment in coating bath with respect to yarn winding speed

| **Motor Speed, V** | **Winding  Speed, cm/s** | **Reaction Time, s**  (10 cm yarn) |
| --- | --- | --- |
| 0.75 | 0.022 | 447 |
| 1.5 | 0.046 | 218 |
| 2.0 | 0.063 | 159 |
| 2.5 | 0.080 | 126 |
| 3.0 | 0.095 | 106 |
| 3.5 | 0.112 | 89 |
| 4.0 | 0.131 | 76 |
| 4.5 | 0.147 | 68 |
| 5.0 | 0.165 | 61 |
| 5.5 | 0.181 | 55 |
| 6.0 | 0.198 | 51 |
| 6.5 | 0.216 | 46 |
| 7.0 | 0.235 | 42 |
| 7.5 | 0.250 | 40 |
| 8.0 | 0.272 | 37 |
| 8.5 | 0.287 | 35 |
| 9.0 | 0.317 | 32 |
| 9.5 | 0.321 | 31 |
| 10.0 | 0.349 | 29 |

Table S2. HR (beats/min) results of ECG trials for Ag and Ag/AgCl e-textile electrodes, measured simultaneously with standard gel electrodes, calculated standard deviation, and coefficient of variation (%) results.

|  | Trial 1 |  | T2 |  | T3 |  | T4 |  | T5 |  |
| --- | --- | --- | --- | --- | --- | --- | --- | --- | --- | --- |
|  | **Gel Ref.** | **Ag e-textile** | **Gel Ref.** | **Ag e-textile** | **Gel Ref.** | **Ag e-textile** | **Gel Ref.** | **Ag e-textile** | **Gel Ref.** | **Ag e-textile** |
| ***Average*** | **56.96** | **56.96** | **57.82** | **57.82** | **55.99** | **55.99** | **58.17** | **58.17** | **58.86** | **58.86** |
| ***Std. Dev.*** | 3.09 | 3.09 | 3.02 | 3.02 | 3.16 | 3.16 | 3.84 | 3.84 | 3.35 | 3.35 |
| ***Var. (%)*** | 5.42 | 5.42 | 5.23 | 5.23 | 5.64 | 5.64 | 6.61 | 6.61 | 5.70 | 5.70 |

|  | Trial 1 |  | T2 |  | T3 |  | T4 |  | T5 |  |
| --- | --- | --- | --- | --- | --- | --- | --- | --- | --- | --- |
|  | **Gel Ref.** | **Ag/AgCl e-textile** | **Gel Ref.** | **Ag/AgCl e-textile** | **Gel Ref.** | **Ag/AgCl e-textile** | **Gel Ref.** | **Ag/AgCl e-textile** | **Gel Ref.** | **Ag/AgCl e-textile** |
| ***Average*** | **57.33** | **57.33** | **57.05** | **57.05** | **57.26** | **57.26** | **56.02** | **56.02** | **55.61** | **55.61** |
| ***Std. Dev.*** | 3.22 | 3.22 | 3.50 | 3.54 | 3.97 | 3.97 | 3.24 | 3.24 | 2.68 | 2.68 |
| ***Var. (%)*** | 5.62 | 5.62 | 6.14 | 6.20 | 6.93 | 6.93 | 5.78 | 5.78 | 4.82 | 4.82 |

Table S3. PCC results for e-textile electrodes compared to standard Ag/AgCl gel electrodes

| **E-textile group** | **Trial 1** | **Trial 2** | **Trial 3** | **Trial 4** | **Average** |
| --- | --- | --- | --- | --- | --- |
| **Ag** | 0.98 | 0.98 | 0.98 | 0.98 | **0.98** |
| **Ag/AgCl** | 0.98 | 0.97 | 0.97 | 0.98 | **0.97** |

1. * Corresponding authors. E-mail address: lekather@student.ubc.ca (Katherine Le); frank.ko@ubc.ca (Frank Ko), peymans@ece.ubc.ca (Peyman Servati). [↑](#footnote-ref-2)
